# Correction: Synthesis and stereocomplex formation of enantiomeric alternating copolymers with two types of chiral centers, poly(lactic acid-*alt*-2-hydroxybutanoic acid)s

**DOI:** 10.1039/d2ra90003c

**Published:** 2022-02-11

**Authors:** Hideto Tsuji, Kazuya Nakayama, Yuki Arakawa

**Affiliations:** Department of Applied Chemistry and Life Science, Graduate School of Engineering, Toyohashi University of Technology Tempaku-cho Toyohashi Aichi 441-8580 Japan ht003@edu.tut.ac.jp

## Abstract

Correction for ‘Synthesis and stereocomplex formation of enantiomeric alternating copolymers with two types of chiral centers, poly(lactic acid-*alt*-2-hydroxybutanoic acid)s’ by Hideto Tsuji *et al.*, *RSC Adv.*, 2020, **10**, 39000–39007. DOI: 10.1039/D0RA08351H.

In the original manuscript, incorrect weight- and number-average molecular weights (*M*_w_ and *M*_n_, respectively) were shown for the synthesized and used polymers in the 2. Experimental section. The correct *M*_w_ and *M*_n_ values of P(LLA-*alt*-l-2HB) and P(DLA-*alt*-d-2HB) were 3.00 × 10^4^, 3.08 × 10^4^, 1.53 × 10^4^, and 1.54 × 10^4^ g mol^−1^, respectively.

Due to the correction of the molecular weight values, the authors would like to remove the following statement regarding the suggested reason for the noncrystallizability of unblended P(LLA-*alt*-l-2HB) and P(DLA-*alt*-d-2HB) in the 3.2. Wide-angle X-ray diffractometry section based on the low molecular weight values: “The noncrystallizability of P(LLA-*alt*-l-2HB) and P(DLA-*alt*-d-2HB) may be due to their lower *M*_w_ values (*M*_w_ = 3.0 × 10^3^ and 3.1 × 10^3^, g mol^−1^, respectively) compared to those of the P(LLA-*co*-l-2HB) (56/44) and P(DLA-*co*-d-2HB) (52/48) random copolymers (*M*_w_ = 1.4 × 10^4^ and 1.6 × 10^4^, g mol^−1^, respectively) and P(LLA-*alt*-GA) and P(DLA-*alt*-GA) alternating copolymers (*M*_w_ = 4.8 × 10^3^ and 5.9 × 10^3^, g mol^−1^, respectively).”.

Due to the same reason, in the 3.3. Differential scanning calorimetry section, the authors would like to remove the statement “The lower *T*_m_ values of P(LLA-*alt*-l-2HB) and P(DLA-*alt*-d-2HB) can be attributable to the low molecular weights compared to those of PLLA and PDLA, P(LLA-*co*-l-2HB) (56/44) and P(DLA-*co*-d-2HB) (52/48), and P(LLA-*alt*-GA) and P(DLA-*alt*-GA).”. Additionally, the authors would like to replace the statement “The *T*_m_ values for P(LLA-*alt*-l-2HB)/P(DLA-*alt*-d-2HB) (*M*_w_ = 3.0 × 10^3^ and 3.1 × 10^3^, g mol^−1^, respectively) blends are higher than those for solvent-evaporated and melt-crystallized (*T*_c_ = 70 °C) P(l-2HB)/P(d-2HB) (*M*_w_ = 1.8 × 10^3^ and 3.3 × 10^3^, g mol^−1^, respectively) homopolymer blends (*T*_m_ = 173.0 and 172.1 °C, respectively)^81^ but lower than those for melt-crystallized (*T*_c_ = 130 °C) PLLA/PDLA (*M*_w_ = 4.0 × 10^3^ and 5.4 × 10^3^, g mol^−1^, respectively) homopolymer blends (*T*_m_ = 197.5 °C),^78^ and solvent-evaporated and melt-crystallized (*T*_c_ = 160 °C) P(LLA-*co*-l-2HB) (56/44)/P(DLA-*co*-d-2HB) (52/48) (*M*_w_ = 1.4 × 10^4^ and 1.6 × 10^4^, g mol^−1^, respectively) *random* copolymer blends (203.6 and 198.4 °C),^63^ and solvent evaporated and melt-crystallized (*T*_c_ = 100 °C) P(LLA-*alt*-GA)/P(DLA-*alt*-GA) (*M*_w_ = 4.8 × 10^3^ and 5.9 × 10^3^, g mol^−1^, respectively) blends (187.8 and 187.6 °C).^67^” with the revised statement “The *T*_m_ values for P(LLA-*alt*-l-2HB)/P(DLA-*alt*-d-2HB) (*M*_w_ = 3.0 × 10^4^ and 3.1 × 10^4^ g mol^−1^, respectively) blends are lower than those for solvent-evaporated and melt-crystallized (*T*_c_ = 70 °C) P(l-2HB)/P(d-2HB) (*M*_w_ = 3.1 × 10^4^ and 3.3 × 10^4^ g mol^−1^, respectively) homopolymer blends (*T*_m_ = 218.9 and 214.5 °C, respectively)^81^ and those for melt-crystallized (*T*_c_ = 130 °C) PLLA/PDLA (*M*_w_ = 4.0 × 10^3^ and 5.4 × 10^3^ g mol^−1^, respectively) homopolymer blends (*T*_m_ = 197.5 °C),^78^ and solvent-evaporated and melt-crystallized (*T*_c_ = 160 °C) P(LLA-*co*-l-2HB) (56/44)/P(DLA-*co*-d-2HB) (52/48) (*M*_w_ = 1.4 × 10^4^ and 1.6 × 10^4^ g mol^−1^, respectively) *random* copolymer blends (203.6 and 198.4 °C)^63^, but similar to those of solvent evaporated and melt-crystallized (*T*_c_ = 100 °C) P(LLA-*alt*-GA)/P(DLA-*alt*-GA) (*M*_w_ = 4.8 × 10^3^ and 5.9 × 10^3^ g mol^−1^, respectively) blends (187.8 and 187.6 °C).^67^”.

The Royal Society of Chemistry apologises for these errors and any consequent inconvenience to authors and readers.

## Supplementary Material

